# Supplementary material for: Small and sick newborn care: Changes in service readiness scoring between baseline and 2023 for 65 neonatal units implementing with NEST360 in Kenya, Malawi, Nigeria, and Tanzania
Source: PLOS Glob Public Health. 2025 Jun 25;5(6):e0004367. doi: 10.1371/journal.pgph.0004367 (PMC12193846; doi:10.1371/journal.pgph.0004367)
Supplement: S1 File — (PDF) [file pgph.0004367.s001.pdf]

Country	Hospital ID	Baseline HFA date	2023 HFA date
Malawi	M001	October 23, 2020	May 31, 2023
Malawi	M002	September 26, 2019	May 23, 2023
Malawi	M003	August 20, 2020	May 23, 2023
Malawi	M004	August 19, 2020	May 29, 2023
Malawi	M005	March 8, 2021	May 29, 2023
Malawi	M006	August 17, 2020	May 26, 2023
Malawi	M007	August 14, 2020	May 22, 2023
Malawi	M008	October 20, 2020	May 25, 2023
Malawi	M009	March 9, 2021	June 6, 2023
Malawi	M010	September 25, 2019	May 24, 2023
Malawi	M011	August 18, 2020	May 30, 2023
Malawi	M012	September 24, 2019	May 24, 2023
Malawi	M013	October 22, 2020	June 5, 2023
Malawi	M014	October 27, 2020	May 30, 2023
Malawi	M015	September 23, 2019	June 6, 2023
Malawi	M016	October 21, 2020	May 22, 2023
Malawi	M017	October 21, 2020	May 29, 2023
Malawi	M018	October 23, 2020	June 5, 2023
Malawi	M019	March 10, 2021	June 2, 2023
Malawi	M020	October 27, 2020	May 26, 2023
Malawi	M021	September 23, 2019	May 26, 2023
Malawi	M022	August 20, 2020	June 2, 2023
Malawi	M023	October 26, 2020	May 25, 2023
Malawi	M024	August 17, 2020	June 5, 2023
Malawi	M025	October 22, 2020	May 25, 2023
Malawi	M026	October 20, 2020	June 6, 2023
Malawi	M027	October 26, 2020	May 22, 2023
Malawi	M028	August 18, 2020	May 30, 2023
Malawi	M029	August 14, 2020	May 23, 2023
Malawi	M030	September 25, 2019	May 24, 2023
Malawi	M031	August 19, 2020	June 1, 2023
Malawi	M032	March 12, 2021	June 7, 2023
Malawi	M033	March 8, 2021	June 2, 2023
Malawi	M034	September 25, 2019	June 1, 2023
Malawi	M035	March 11, 2021	May 31, 2023
Malawi	M036	September 24, 2019	June 1, 2023
Kenya	K017	February 10, 2021	July 11, 2023
Kenya	K040	September 29, 2020	July 5, 2023
Kenya	K041	September 28, 2020	July 4, 2023
Kenya	K044	September 17, 2020	July 6, 2023
Kenya	K051	February 16, 2021	July 4, 2023
Kenya	K052	September 21, 2020	July 10, 2023
Kenya	K053	December 4, 2019	July 3, 2023

Kenya	K055	September 23, 2020	July 6, 2023
Kenya	K058	December 2, 2019	July 5, 2023
Kenya	K063	September 14, 2020	July 10, 2023
Kenya	K071	March 17, 2021	July 7, 2023
Kenya	K072	February 8, 2021	July 3, 2023
Kenya	K076	September 16, 2020	July 7, 2023
Tanzania	T001	October 19, 2020	May 17, 2023
Tanzania	T002	February 8, 2021	May 30, 2023
Tanzania	T003	February 4, 2021	June 2, 2023
Tanzania	T004	October 22, 2020	May 26, 2023
Tanzania	T005	October 23, 2020	May 25, 2023
Tanzania	T006	October 21, 2020	May 24, 2023
Tanzania	T007	October 20, 2020	May 23, 2023
Nigeria	N001	September 1, 2020	June 26, 2023
Nigeria	N002	September 1, 2020	July 3, 2023
Nigeria	N003	March 11, 2021	July 4, 2023
Nigeria	N004	March 10, 2021	June 27, 2023
Nigeria	N005	March 12, 2021	June 23, 2023
Nigeria	N006	March 11, 2021	June 30, 2023
Nigeria	N007	September 1, 2020	June 26, 2023
Nigeria	N008	September 1, 2020	July 3, 2023
Nigeria	N009	March 11, 2021	July 4, 2023
Nigeria	N010	March 10, 2021	June 27, 2023
Nigeria	N011	March 12, 2021	July 5, 2023
