# Supplementary material for: Small and sick newborn care: Changes in service readiness scoring between baseline and 2023 for 65 neonatal units implementing with NEST360 in Kenya, Malawi, Nigeria, and Tanzania
Source: PLOS Glob Public Health. 2025 Jun 25;5(6):e0004367. doi: 10.1371/journal.pgph.0004367 (PMC12193846; doi:10.1371/journal.pgph.0004367)
Supplement: S2 File — (DOCX) [file pgph.0004367.s002.docx]

**S2 File**: Standards-based and level-2+ clinical interventions and sub-modules included in Health Facility Assessment (HFA) scoring.

| **Standards-based Health System Building Blocks (HSBBs) with sub-modules** | **Level-2+ clinical interventions with sub-modules** |
| --- | --- |
| 1. **Medical devices and supplies**  - Medical device requirements - Laboratory - Pharmacy - Biomedical workshop  1. **Human resources**  - People - Education - Enabling environment  1. **Infrastructure**  - Electrical power - Medical gases and vacuum - Referral - Space and design - Water, sanitation, and hygiene  1. **Information systems**  - Data collection - Data management - Maternal and perinatal death surveillance and response (MPDSR) - Foundations for information systems  1. **Family centred care**  - Organisation of care - Discharge and early development - Parent power - Kangaroo mother care (KMC)  1. **Governance and leadership**  - Hospital management | 1. **Thermal care including - Kangaroo Mother Care (KMC) for all stable neonates <2000 grams**   *Diagnosis*   - Temperature monitoring for baby   *Treatment/management*   - Items for thermal support - Kangaroo Mother Care (KMC) - Devices for thermal support - Device power sources - Infection prevention - Infrastructure for thermal support - Guidelines, initiation of care, and training  1. **Assisted feeding and intravenous fluids**   *Diagnosis*   - Blood glucose screening   *Treatment/management*   - Breast feeding and milk banking - Cup feeding - Nasogastric tube feeding - Intravenous fluids - Intravenous fluids equipment and consumables - Device power sources - Infection prevention - Guidelines, initiation of care, and training  1. **Safe administration of oxygen**   *Diagnosis*   - Oxygen assessment - Vital sign monitoring   *Treatment/management*   - Items for oxygen provision - Oxygen sources, including devices - Device power sources - Infection prevention - Guidelines, initiation of care, and training  1. **Detection and management of neonatal sepsis with injection antibiotics**   *Diagnosis*   - Readiness for culture - Temperature monitoring for baby   *Treatment/management*   - Antibiotics - Guidelines, initiation of care, and training  1. **Detection and management of neonatal jaundice with phototherapy**   *Diagnosis*   - Bilirubin measurement - Laboratory can assess underlying causes - Other monitoring for baby   *Treatment/management*   - Equipment for phototherapy provision - Consumables for phototherapy provision - Device power sources - Therapeutic irradiance - Infection prevention - Guidelines, initiation of care, and training  1. **Detection and management of neonatal encephalopathy**   *Diagnosis*   - Diagnostics   *Treatment/management*   - Seizure management - Therapeutic hypothermia - Guidelines, initiation of care, and training  1. **Detection and referral/management of congenital abnormalities**   *Treatment/management*   - Referral communication systems - Referral transport systems - Guidelines, initiation of care, and training  1. **Continuous Positive Airway Pressure (CPAP) management of preterm resp. distress**   *Diagnosis*   - Oxygen assessment - Vital sign monitoring   *Treatment/management*   - Items for Continuous Positive Airway Pressure (CPAP) - Equipment for Continuous Positive Airway Pressure (CPAP) - Oxygen sources, including devices - Device power sources - Infection prevention - Guidelines, initiation of care, and training  1. **Perform exchange transfusion for a newborn**   *Diagnosis*   - Bilirubin measurement - Laboratory can assess underlying causes - Other monitoring for baby   *Treatment/management*   - Equipment for exchange transfusion - Blood bank support for transfusion - Infection prevention - Guidelines, initiation of care, and training  1. **Provide follow-up of at-risk newborns***   *Treatment/management*   - Guidelines and discharge plan |

**Note**: *Interventions with fewer than 5 items were excluded from overall level-2+ scores. Content adapted from Penzias et al. “Quantifying health facility service readiness for small and sick newborn care: comparing standards-based and WHO level-2+ scoring for 64 hospitals implementing with NEST360 in Kenya, Malawi, Nigeria, and Tanzania” (*BMC Pediatrics* 2024; 23:656, https://pubmed.ncbi.nlm.nih.gov/38475761/).
