# Supplementary material for: Small and sick newborn care: Changes in service readiness scoring between baseline and 2023 for 65 neonatal units implementing with NEST360 in Kenya, Malawi, Nigeria, and Tanzania
Source: PLOS Glob Public Health. 2025 Jun 25;5(6):e0004367. doi: 10.1371/journal.pgph.0004367 (PMC12193846; doi:10.1371/journal.pgph.0004367)
Supplement: S3 File — (DOCX) [file pgph.0004367.s003.docx]

**S3 File**: Local ethical approval for the complex evaluation of the implementation of a small and sick newborn care package with Newborn Essential Solutions and Technologies (NEST360).

| **Country** | **Protocol Title** | **Protocol ID** |
| --- | --- | --- |
| **Kenya** | Using a Health Facility Assessment to Assess Quality of New Born Care in Kenya | MSU/DRPI/MUERC/00810/19 |
|  | Evaluating the effects of technology and workforce enhancement to support neonatal hospital care in Kenya. | KEMRI/RES/7/3/1 |
|  | The Impact of NEST360 Interventions on Quality of Neonatal Care and Changes in Health Systems Resources on Neonatal Outcomes in Kenyan Health Facilities | MSU/DRPI/MUSERC/01200/23 |
| **Malawi** | Using a Health Facility Assessment to Assess Quality of Newborn Care in Malawi | NHSRC 2463 |
| **Nigeria** | Quality Improvement Study of the Implementation of a Package of Trainings and Technologies for the Delivery of Comprehensive Newborn Care in Nigeria: A Multi-Country Study | **LUTH:** ADM/DCST/HREC/APP/3487 |
|  |  | **UCH:** UI/EC/20/0713 |
|  |  | **NHREC:** NHREC/01/01/2007 |
| **Tanzania** | Implementation study to improve the quality of comprehensive newborn care through introduction of the package of Newborn Essential Solutions and Technologies (NEST) in Tanzania | **IHI:**IHI/IRB/01-2021 |
|  |  | **MUHAS:**MUHAS-REC-12-2019-072 |
|  |  | **NIMR:** 3405 |
| **USA** | Using a Health Facility Assessment to Assess Quality of New Born Care in Kenya | IRB-FY2020-266 |
|  | Quality Improvement Study of the Implementation of a Package of Trainings and Technologies for the Delivery of Comprehensive Newborn Care in Nigeria: A Multi-Country Study | IRB-FY2020-271 |
|  | Implementation study to improve the quality of comprehensive newborn care through introduction of the package of Newborn Essential Solutions and Technologies (NEST) in Tanzania | IRB-FY2020-237 |

**Abbreviations**: MSU; Michigan State University, DRPI; Disability Right Promotion International, MUERC; Maseno University Ethics Review Committee, NHSRC; National Health Science Research Committee, LUTH; Lagos University Teaching Hospital, UCH; University College Hospital, NHREC; National Health Research Ethics Committee, IHI; Ifakara Health Institute, MUHAS; Muhimbili University of Health and Allied Science, NIMR; National Institute for Medical Research, IRB; Institutional Review Board
