# Supplementary material for: Small and sick newborn care: Changes in service readiness scoring between baseline and 2023 for 65 neonatal units implementing with NEST360 in Kenya, Malawi, Nigeria, and Tanzania
Source: PLOS Glob Public Health. 2025 Jun 25;5(6):e0004367. doi: 10.1371/journal.pgph.0004367 (PMC12193846; doi:10.1371/journal.pgph.0004367)
Supplement: S4 File — (DOCX) [file pgph.0004367.s004.docx]

**S4 File**: Hospital level and admissions for 65 neonatal units implementing with Newborn Essential Solutions and Technologies (NEST360) at in Kenya, Malawi, Nigeria, and Tanzania.

|  | Overall | Malawi | Kenya | Tanzania | Nigeria |
| --- | --- | --- | --- | --- | --- |
| Neonatal units | 65 | 36 | 13 | 7 | 9 |
| Median annual admissions | 1050  [IQR 582-1971] | 963  [IQR 609-1338] | 2100  [IQR 1176-2454] | 2802  [IQR 1710-4104] | 324  [IQR 246-1026] |
| Level | | | | | |
| Tertiary/ Secondary | 35 | 6 | 13 | 7 | 9 |
| Primary | 30 | 30 | 0 | 0 | 0 |
| Admissions per year (number of hospitals) | | | | | |
| <1100 | 36 | 24 | 3 | 0 | 9 |
| >1100 | 29 | 12 | 10 | 7 | 0 |
