# Supplementary figures and images for: Small and sick newborn care: Changes in service readiness scoring between baseline and 2023 for 65 neonatal units implementing with NEST360 in Kenya, Malawi, Nigeria, and Tanzania
Source: PLOS Glob Public Health. 2025 Jun 25;5(6):e0004367. doi: 10.1371/journal.pgph.0004367 (PMC12193846; doi:10.1371/journal.pgph.0004367)

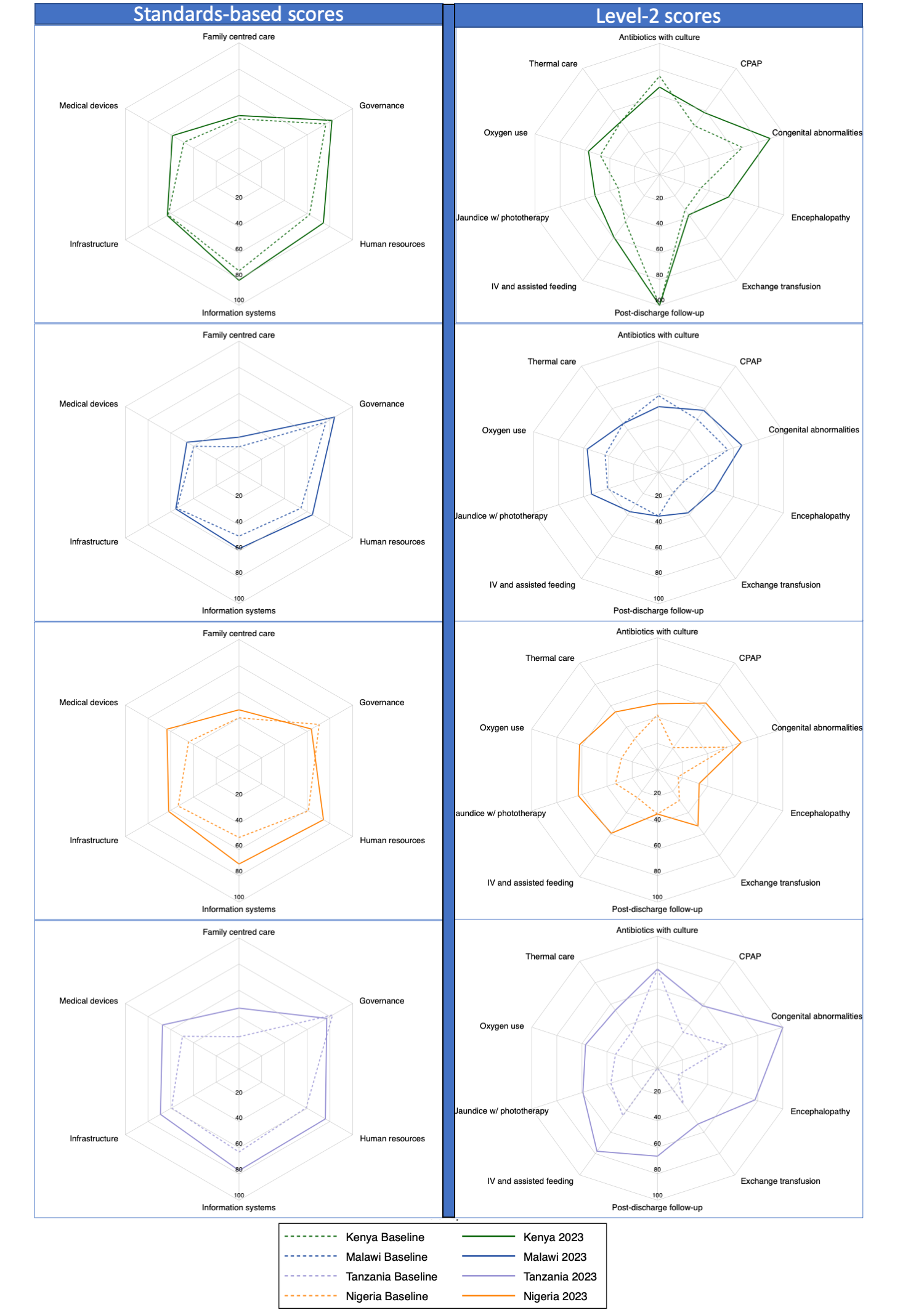

Supplement: S5 File — Legend: Abbreviations: IV – Intravenous; CPAP – Continuous Positive Airway Pressure. (TIF) [file pgph.0004367.s005.tif]
