# Supplementary material for: Small and sick newborn care: Changes in service readiness scoring between baseline and 2023 for 65 neonatal units implementing with NEST360 in Kenya, Malawi, Nigeria, and Tanzania
Source: PLOS Glob Public Health. 2025 Jun 25;5(6):e0004367. doi: 10.1371/journal.pgph.0004367 (PMC12193846; doi:10.1371/journal.pgph.0004367)
Supplement: Attachment — Submitted filename: Response to Reviewers.docx [file pgph.0004367.s007.docx]

*14^th^ February 2025*

Dear Editorial team,

***Re:*** *"Small and sick newborn care: Changes in service readiness scoring between baseline and 2023 for 63 hospitals implementing with NEST360 in Kenya, Malawi, Nigeria, and Tanzania " (PGPH-D-24-01799)*

Thank you for the opportunity to submit a revised version of our manuscript. We are deeply appreciative to the editor and reviewers for their thoughtful comments and suggestions. These specific comments enabled us to improve and strengthen the manuscript. Please find below our line-by-line response to the highlighted journal requirements and reviewer’s comments (journal requirements and reviewer comments are in italics).

Thank you for your consideration of this re-submission and please let us know if more information is required.

Yours sincerely,

Rebecca Penzias (on behalf of the team)

*Rebecca E. Penzias, BA MSc PhD*

*Maternal, Adolescent, Reproductive & Child Health (MARCH) Centre*

*London School of Hygiene & Tropical Medicine, Keppel Street (LSHTM)*

*London, WC1E 7HT, United Kingdom*

E: rebecca.penzias@lshtm.ac.uk

**SPECIFIC JOURNAL REQUIREMENTS RESPONSES**

***Comment 1:*** *Please include a complete copy of PLOS’ questionnaire on inclusivity in global research in your revised manuscript. Our policy for research in this area aims to improve transparency in the reporting of research performed outside of researchers’ own country or community. The policy applies to researchers who have travelled to a different country to conduct research, research with Indigenous populations or their lands, and research on cultural artefacts. The questionnaire can also be requested at the journal’s discretion for any other submissions, even if these conditions are not met. Please find more information on the policy and a link to download a blank copy of the questionnaire here:* [*https://journals.plos.org/globalpublichealth/s/best-practices-in-research-reporting*](https://url.uk.m.mimecastprotect.com/s/YO-gC8EVLUlN2ZWInfYIysTQi?domain=journals.plos.org)*. Please upload a completed version of your questionnaire as Supporting Information when you resubmit your manuscript.*

***Response: Thank you. We have added the PLOS’ questionnaire on inclusivity in global research as a supplemental file with the submission.***

***Editorial comment 2:*** *Please amend your detailed Financial Disclosure statement. This is published with the article. It must therefore be completed in full sentences and contain the exact wording you wish to be published.*

***Please only choose the relevant sentences from below***

*1. Please clarify all sources of funding (financial or material support) for your study. List the grants (with grant number) or organizations (with url) that supported your study, including funding received from your institution. 
2. State the initials, alongside each funding source, of each author to receive each grant.
3. State what role the funders took in the study. If the funders had no role in your study, please state: “The funders had no role in study design, data collection and analysis, decision to publish, or preparation of the manuscript.”
4. If any authors received a salary from any of your funders, please state which authors and which funders*

*If you did not receive any funding for this study, please simply state: “The authors received no specific funding for this work.”*

***Response:*** ***Thank you. Please see detailed financial disclosure statement below:***

***This work is funded through the NEST360 alliance with thanks to John D. and Catherine T. MacArthur Foundation, the Bill & Melinda Gates Foundation, ELMA Philanthropies, The Children’s Investment Fund Foundation UK, The Lemelson Foundation, The Sall Family Foundation, and the Ting Tsung and Wei Fong Chao Foundation under agreements to William Marsh Rice University as the prime grant holder under sub agreement to LSHTM. The funders had no role in the study design, data collection and analysis, decision to publish, or manuscript preparation.***

***Editorial comment 3:*** *Please send a completed 'Competing Interests' statement, including any COIs declared by your co-authors. If you have no competing interests to declare, please state "The authors have declared that no competing interests exist". Otherwise please declare all competing interests beginning with the statement "I have read the journal's policy and the authors of this manuscript have the following competing interests:*

***Response: Thank you. Please see detailed ‘Competing Interests’ statement below:***

***The authors have declared that no competing interests exist.***

***Editorial comment 4:*** *In the online submission form, you indicated that "All partners in the NEST360 alliance collaborated to create and sign data sharing and transfer agreements. The dataset from this study will be accessible upon request, pending approval from the NEST360 learning network and collaborating parties.".*

*All PLOS journals now require all data underlying the findings described in their manuscript to be freely available to other researchers, either 
1. In a public repository, 
2. Within the manuscript itself, or 
3. Uploaded as supplementary information.*

*This policy applies to all data except where public deposition would breach compliance with the protocol approved by your research ethics board. If your data cannot be made publicly available for ethical or legal reasons (e.g., public availability would compromise patient privacy), please explain your reasons by return email and your exemption request will be escalated to the editor for approval. Your exemption request will be handled independently and will not hold up the peer review process, but will need to be resolved should your manuscript be accepted for publication. One of the Editorial team will then be in touch if there are any issues.*

***Response: Thank you for noting this. The NEST360 program is ongoing and the main analyses is an impact evaluation of the NEST360 package in reducing neonatal mortality. The impact evaluation will be conducted in 2026 and therefore, we are unable to share data at this stage due to the pending final analysis of the main outcome of the program. As noted, the NEST360 Alliance is committed to making data accessible through the collaborative learning network with the hosting of the data in an African institution. At this stage, we will pursue the exemption request by email to the editor.***

***Editorial comment 5:*** *Please provide an Author Summary. This should appear in your manuscript between the Abstract (if applicable) and the Introduction, and should be 150–200 words long. The aim should be to make your findings accessible to a wide audience that includes both scientists and non-scientists. Sample summaries can be found on our website under Submission Guidelines:*

[*https://journals.plos.org/globalpublichealth/s/submission-guidelines#loc-parts-of-a-submission*](https://url.uk.m.mimecastprotect.com/s/NlSTC9gWMIv4lgAuEh2Iq4U_m?domain=journals.plos.org)

***Response: Thank you. We have included an author summary in lines 93-117 as below:***

***Why Was This Study Done?***

- ***Small and sick newborns face significant risks of death and disability, and hospitals must be ready to provide specialised care.***
- ***Tools to assess and track readiness to provide care for small and sick newborns, and standardised methods to evaluate progress over time are needed.***
- ***This study aimed to address these gaps by developing and applying methodologies to evaluate change in service readiness to provide small and sick newborn care.***

***What Did the Researchers Do and Find?***

- ***We collected data at two time points from 65 neonatal units in Kenya, Malawi, Nigeria, and Tanzania using the NEST360/UNICEF Health Facility Assessment tool between 2019 and 2023.***
- ***Two methods of scoring service readiness were used: one using health system standards (World Health Organization building blocks) and another focused on clinical interventions for small and sick newborn care.***
- ***Median service readiness scores improved from 41% to 55% (14% increase) for clinical intervention scores and from 51% to 60% (9% increase) for standards-based scores. Hospitals in Tanzania and Nigeria showed the greatest improvements.***

***What Do These Findings Mean?***

- ***These findings highlight the feasibility of using standardised tools and methodologies to track service readiness for small and sick newborn care.***
- ***Improvements in readiness scores indicate progress, but gaps remain, particularly in countries with slower rates of change.***

***These findings can guide policymakers, health providers, and researchers to prioritise investments and interventions to meet global newborn survival targets.***

***Editorial comment 6:*** *We have noticed that you have uploaded Supporting Information files, but you have not included a list of legends. Please add a full list of legends for your Supporting Information files after the references list.*

***Response:*** ***Thank you. We have uploaded additional files at the request of the journal, including: i) PLOS human subjects research checklist, ii) ethical approvals and waivers, and iii) protocol documents. However, we do not wish these to be published as supporting information, so have not included them in the legend for supporting information files. We are happy to include the data collection dates by neonatal unit as supplemental information, and have revised the legend accordingly in lines 696-716 as below:***

***S1 Additional File****: Baseline and 2023 Health Facility Assessment (HFA) data collection dates by hospital and country.*

***S2 Additional File****: Standards-based and level-2+ clinical interventions and sub-modules included in Health Facility Assessment (HFA) scoring.*

***S3 Additional file****: Local ethical approval numbers for the complex evaluation of the implementation of a small and sick newborn care package with Newborn Essential Solutions and Technologies (NEST360).*

***S4 Additional file****: Inclusivity in global research checklist.*

***S5 Additional file****: Hospital level and admissions for 65 neonatal units implementing with Newborn Essential Solutions and Technologies (NEST360) at in Kenya, Malawi, Nigeria, and Tanzania.*

***S6 Additional file****: Spider plot of median standards-based scores by health system building blocks and level-2+ scores by clinical intervention for 65 neonatal units implementing with Newborn Essential Solutions and Technologies (NEST360) at baseline and 2023 follow-up with separate country-specific panels. Legend: Abbreviations: IV – Intravenous; CPAP – Continuous Positive Airway Pressure*

**SPECIFIC REVIEW RESPONSES**

**REVIEWER #1**

***General comments****: Thank you for asking me to review this well-written paper on changes in service readiness for small and sick newborns (SSNC). I have a few suggestions for the authors:*

***Response: Thank you for reviewing the manuscript. We are appreciative of your insights and thoughtful comments which have helped to improve the paper. We have provided a response to all your comments point by point as below.***

***Comment 1***: *It is difficult to fully understand the paper without reading your other 2 papers - reference 7 & 8. I think a neutral reader should be able to pick up this paper and read to understand without having to visit the other papers. A few suggestion to improve on this –*

*a. line 90 and 91. Maybe a few words on the scoring approaches for some background. I assume these are based on the HSBB and level 2+ clinical intervention. With the way the paper is currently written, there is no introduction to this before they come up in the aims. This makes your aims difficult to understand without reading your 2 papers or going into reading your methodology

b. The HFA can be better described - how many domains/ questions does it assess? I found this in another paper. The authors speak about modules and sub-modules later in the methods. A gentle introduction on how the HFA was structured will allow the reader understand this without having to visit the other linked papers.*

***Response: Thank you for your comment and suggestions to include additional details to the paper to make it stand alone without having to visit the other papers. We have included your suggestions in the main paper as below.***

- *line 90 and 91. Maybe a few words on the scoring approaches for some background. I assume these are based on the HSBB and level 2+ clinical intervention. With the way the paper is currently written, there is no introduction to this before they come up in the aims. This makes your aims difficult to understand without reading your 2 papers or going into reading your methodology.*

***Response: We have included additional detail about the two scoring approaches in the introduction to provide more background on the scoring methodology ahead of the aims. This has been revised in lines 136-40 as below:***

***Two scoring approaches were developed to quantify HFA data for SSNC: a) standards-based, including items for SSNC service readiness by health system building block (HSBB), and scored on availability and functionality, and b) level-2+, scoring items on readiness to provide WHO level-2 + clinical interventions.***

- *The HFA can be better described - how many domains/ questions does it assess? I found this in another paper. The authors speak about modules and sub-modules later in the methods. A gentle introduction on how the HFA was structured will allow the reader understand this without having to visit the other linked papers.*

***Response: We agree that this was unclear, and have revised the background about the HFA tool to include additional detail about the number of items and modules within the tool. This has been revised in lines 190-93 as below:***

***The tool includes 1508 items across ten discrete modules and are organised into adapted HSBBs: infrastructure, medical devices and supplies, human resources, information systems, family centred care, and governance.***

**In addition, we have revised the HFA scoring methodology section to clarify how each score was organised into modules and sub-modules. We have also added more detail about the number of items from the tool included in each score. This has been revised in lines 210-24 as below:**

***Two summary scoring approaches were developed to quantify service readiness for SSNC: standards-based, and level-2+ scoring.(8) Standards-based scores were organised by the HSBB framework adapted from WHO, and include all items required for SSNC service readiness, and were scored on availability and functionality.(17) Within each HSBB, items were organised into HSBB sub-modules according to content area (i.e. education within the human resources HSBB). Level-2+ scores were organised by the ten WHO level-2+ clinical interventions, and were scored based on readiness to provide basic, comprehensive, or gold-standard care for that intervention.(2) Within each clinical intervention, items were organised into intervention sub-modules to include all potential pathways for diagnosis/screening and treatment/management for that intervention (i.e. cup feeding and nasogastric tube feeding within the assisted feeding intervention). Standards-based scores focus on a health systems approach and assess service readiness across the health system, and include 1043 (69%) of the 1508 HFA items. The level-2+ scores focus specifically on interventions provided on the neonatal unit, and as such, include fewer items with 309 (20%) of 1508 items scored (Additional File 2).***

***Comment 2:*** *For such a detailed HFA completed in under 24 hours - how and to who was this administered and what methods were used? The questions span what nurses or lab manager would know and seems to cut across multiple hospital departments linked to SSNC.*

***Response: Thank you for noting this. The HFA is completed by a multidisciplinary team of seven assessors who collected data across the hospital. We have expanded on this in the manuscript to provide additional detail on the HFA methodology and who the tool was administered to. This has been revised in lines 198-203 as below:***

***HFA data collection was completed by a team of seven multidisciplinary assessors in one day at each hospital using observation and interviewer-led assessment with hospital staff, including nurses, biomedical technicians, laboratory managers, hospital management, and other relevant department staff, and collecting data on a mobile REDCap application on Android tablets.***

***Comment 3:*** *Results speak about 65 neonatal units but title and abstract talk about 63. why the discrepancy?*

***Response: Thank you for noting the discrepancy. Two hospitals in Nigeria have separate neonatal units for inborn and outborn babies. These are genuine separate wards in different buildings across the hospital. We have conducted our analysis at the neonatal unit level. We understand this discrepancy may add confusion for the reader and have removed reference to the 63 hospitals throughout the paper and we refer to the 65 neonatal units throughout the paper.***

***Comment 4:*** *Please in the methods, highlight the continuous quality improvement linked to NEST-360, so the reader appreciates the intervention was not one-off and was sustained over a period of time.*

***Response: Thank you for this suggestion. We have revised the NEST360 methodology section to include a reference to continuous quality improvement. This has been revised in lines 177-82 as below:***

***NEST360 particularly focuses on providing a bundle of innovative technologies with training and mentoring for clinicians and biomedical technicians, strengthening information systems, and implementing other evidence-based strategies for high-quality and sustainable newborn care, including government-led continuous quality improvement (QI). Quarterly QI visits began in 2021 with an average of three visits per year at each hospital.***

***Comment 5:*** *Was the decision to analyse Malawi as low volume and high volume determined a priori or this was after a look at the data? The authors should be transparent about this in the methods*

***Response: Thank you for this suggestion. We decided a priori to disaggregate hospitals by hospital-level and volume of admissions. However, after exploration of the data, we decided only to report disaggregated scores for hospitals in Malawi given the large number of hospitals in Malawi and implementation at scale throughout the country. We have revised the manuscript to clarify this in lines 248-52 as below:***

***We also chose a priori to disaggregate scores by hospital-level (tertiary, secondary, and primary) and annual number of admissions. After exploration, we decided to report scores disaggregated by hospital-level and admissions for Malawi only given the large number of hospitals in Malawi and implementation in all districts throughout the country.***

***Comment 8:*** *Although human resources had the highest improvements between periods, this was driven by education & training and not an increase in people. The authors should reflect on this in the discussion including any possible implications. Currently there is some reflection on what was not measured (nurse to patient ratios), but you have a whole subdomain on people in the HSBB which did not change much between the baseline and after NEST.*

***Response: Thank you for highlighting this. We agree that it is important to reflect on the lack of change in the ‘people’ subdomain of human resources, noting this highlights urgent need for investment in additional staff. We have revised this in lines 458-69 as below:***

***Human resources, particularly education and training for clinicians, biomedical technicians, and data clerks, was also supported by the NEST360 programme.(21) Jointly with government partners, NEST360 teams led generic instructor courses (GICs) to expand availability of high-quality trainers for clinical and biomedical education in countries implementing with NEST360.(22) Although there were improvements in the human resources scores, the standards-based scores focused on people, education, and enabling environment, and did not include staff-to-patient ratios, which currently have no standards, and remain a challenge across hospitals in the four countries. In addition, the people sub-module, which focuses on staffing allocation and staff numbers, did not improve, which suggests the need for additional government investment to strengthen the neonatal health workforce by recruiting, training, and retaining neonatal unit staff and supporting cadres, including clinical specialists.***

**REVIEWER #2**

***General comments****: Thank you for submitting this well-written manuscript on an important and timely topic related to the global neonatal survival target and Every Newborn Action Plan.*

**Response: Thank you for reviewing the manuscript. We are appreciative of your insights and thoughtful comments which have helped to improve the paper. We have provided a response to all your comments as below.**

***Comment 1:*** *The study aim(s) are clearly stated (lines 113-118) as (i) evaluating changes in standards-based and level-2+ service readiness between baseline (2019-2021) and 2023 follow-up scores for each neonatal unit according to Health System Building Block (HSBB) or clinical intervention, and (ii) evaluating changes in standards-based and level-2+ service readiness for each neonatal unit according to HSBB or clinical intervention disaggregated by sub
module scores.

It seems as though an additional aim of this study was to explore factors that contributed to (or perhaps were barriers to) improvements in service readiness as measured by the HFA score. On line 360, you state: "Tracking changes and understanding the drivers of change in service readiness are vital." The "Discussion" section of your manuscript also explores various drivers of change in service readiness.

If possible, I suggest that you clearly state an additional aim, "Exploring the drivers of change in service readiness," as it appears that this was one of the aims of your research. Adding a subsection on the drivers of change in service readiness would allow you to clearly describe (perhaps country by country) potential drivers of change in the HFA service readiness score.

Adding this additional explicit aim, which seems to be implicitly part if this research, and more discussion on the drivers of change, could strengthen the applicability and usefulness of this important research.*

***Response: Thank you for your suggestion to add an explicit aim exploring the drives of change in service readiness.***

***The first aim of the paper is to ‘evaluate change between baseline and 2023 in standards-based and level-2+ service readiness scores and by HSBB or clinical intervention, overall and by country’. This aim focuses on evaluating changes in overall scores and scores by module. The second aim of the paper is to ‘evaluate change in standards-based and level-2+ service readiness sub-module scores for SSNC at baseline and 2023 follow-up overall and by country’. This second aim focuses on evaluating the drivers of change for the standards-based and level-2+ scores by analysing the changes in sub-module scores to understand what categories contribute to changes in the module scores. Though this objective focuses on the drivers of score changes over time, we prefer to frame the objective as highlighting change in sub-module scores to avoid confusing drivers of score change with drivers of change within the hospital.***
